# Hypothesis-Generating Analysis of Four Immunosuppressive Regimens After Simultaneous Pancreas-Kidney Transplantation Using the United States Food and Drug Administration Adverse Event Reporting System

**DOI:** 10.7759/cureus.107193

**Published:** 2026-04-16

**Authors:** Toru Ogura, Chihiro Shiraishi, Aiko Urawa

**Affiliations:** 1 Clinical Research Support Center, Mie University Hospital, Tsu, JPN; 2 Department of Pharmaceutical Sciences for Health Crisis Management, Faculty of Pharmaceutical Sciences, Fukuoka University, Fukuoka, JPN; 3 Organ Transplantation Center, Mie University Hospital, Tsu, JPN

**Keywords:** adverse events, immunosuppressive regimens, pharmacovigilance, safety signal detection, simultaneous pancreas–kidney transplantation

## Abstract

Background

Simultaneous pancreas-kidney (SPK) transplantation is a key treatment option for patients with diabetes and end-stage renal disease, but intensive multidrug immunosuppression increases the risk of adverse events (AEs). Evidence guiding stepwise de-escalation of immunosuppressive regimens in this setting remains limited, as prospective studies are difficult to conduct due to the rarity and complexity of the procedure. Large postmarketing pharmacovigilance databases such as the United States Food and Drug Administration Adverse Event Reporting System (FAERS) provide an opportunity to evaluate the safety of immunosuppressive strategies in real‑world practice.

Objectives

To characterize exploratory AE reporting patterns across four post-transplant immunosuppressive regimens after SPK transplantation.

Methods

AE reports for SPK transplant recipients were retrieved from the FAERS database (2004Q1-2025Q2). Immunosuppressive exposure was classified into four regimen groups: tacrolimus, mycophenolate mofetil, prednisone, and basiliximab (TMPB); tacrolimus, mycophenolate mofetil, and prednisone (TMP); tacrolimus and mycophenolate mofetil (TM); and tacrolimus monotherapy (T). Reports involving additional immunosuppressive agents were excluded. Transplant rejection, infections, and other clinically relevant AEs were grouped into predefined categories, and serious AEs (SAEs) were identified according to regulatory criteria. Reporting proportions were calculated for each AE category, and reporting odds ratios (RORs) and adjusted RORs (aRORs) were estimated using the TMPB group as the reference, with adjustment for sex, age, and continent. A Bonferroni‑adjusted two‑sided significance level of 0.017 was applied to account for three primary between‑regimen comparisons.

Results

A total of 569 SPK recipients met the inclusion criteria (TMPB group, *N* = 59; TMP group, *N* = 231; TM group, *N* = 95; T group, *N* = 184). Reporting of transplant rejection was highest in the TMPB group and lower in the TMP, TM, and T groups; all three regimens had lower RORs and aRORs for rejection than TMPB. Infections were reported more frequently in the TMPB and TMP groups and less frequently in the TM and T groups, with lower adjusted odds in the TM and T groups versus TMPB. Reporting proportions for renal, pancreatic, gastrointestinal, hematologic disorders, and drug toxicity were broadly similar across regimens. SAEs were numerically more frequent in the T group than in the other regimens, but this difference did not meet the prespecified significance threshold. Analyses in kidney‑only transplant recipients yielded qualitatively similar trends.

Conclusions

Across four post-transplant immunosuppressive regimens, lower reporting of transplant rejection and infections was observed in the TM group; however, because regimen assignment likely reflects post-transplant phase and clinical stability, these patterns should be interpreted as exploratory reporting differences rather than comparative safety effects. Because spontaneous reporting data cannot adequately adjust for time-related confounding, these hypothesis-generating findings warrant prospective validation.

## Introduction

Simultaneous pancreas-kidney (SPK) transplantation is an established treatment option for patients with diabetes and end‑stage renal disease, offering improved glycemic control and renal function compared with solitary pancreas or kidney transplantation alone [1]. Since the first pancreas transplant was performed in 1966, >60,000 pancreas transplants have been conducted worldwide, and over 70% of these have involved SPK transplantation [2]. Despite these clinical benefits, only about 2,000 pancreas transplants are performed globally each year, whereas kidney transplantation alone accounts for the vast majority of solid organ transplants [2]. This discrepancy reflects the surgical complexity and heightened immunologic burden associated with dual‑organ transplantation, which are linked to higher rates of transplant rejection and infection than those observed after kidney transplantation alone [3,4]. To mitigate these risks, intensive combination immunosuppressive regimens are routinely employed, most commonly including tacrolimus, mycophenolate mofetil, prednisone, and basiliximab, which exert complementary effects through distinct mechanisms [5]. The risk of rejection and infection is greatest in the early post‑transplant period, necessitating strong immunosuppression during this phase. In clinical practice, immunosuppressive therapy is often initiated with multidrug therapy and then gradually tapered over time to balance graft function and adverse events (AEs). This transition may proceed from a basiliximab-containing regimen to triple therapy without basiliximab, followed by dual therapy with tacrolimus and mycophenolate mofetil, and in some cases to tacrolimus monotherapy as a subsequent simplified regimen [5].

Although such stepwise de‑escalation of immunosuppression is widely adopted, evidence supporting the comparative safety of specific immunosuppressive regimens and tapering strategies after SPK transplantation remains limited. Prospective randomized trials are difficult to conduct because SPK transplantation is relatively rare and raises ethical and logistical challenges. As a result, current practice is often guided by extrapolation from kidney-only transplantation and by center-specific experience, and there is uncertainty about the extent to which multidrug regimens can be simplified while maintaining acceptable safety. In particular, it remains unclear whether the regimens corresponding to later simplification stages, including dual therapy with tacrolimus and mycophenolate mofetil and tacrolimus monotherapy, are associated with meaningful differences in rejection, infection, and serious AEs (SAEs) in real-world settings.

Large postmarketing pharmacovigilance databases offer one means to explore these questions by characterizing patterns of AE reporting under different immunosuppressive regimens in routine clinical practice. The United States Food and Drug Administration Adverse Event Reporting System (FAERS) [6] collects spontaneous reports of suspected drug‑related AEs from multiple countries and care settings, and thus can provide complementary information on the safety profiles of immunosuppressive strategies that are difficult to evaluate in conventional trials. At the same time, such data have important limitations, including underreporting, reporting bias, and incomplete clinical details, and therefore are better suited to generating hypotheses than establishing causal effects. The aim of this study was to characterize AE reporting patterns across four post-transplant immunosuppressive regimens after SPK transplantation using FAERS and generate exploratory safety signals that may help inform future prospective and population‑based studies in this population.

## Materials and methods

Data source

Data were obtained from FAERS, a pharmacovigilance database updated quarterly since 2004 (data current as of September 29, 2025). FAERS was known as the Adverse Event Reporting System (AERS) between the first quarter of 2004 (2004Q1) and 2012Q3 (file names: aers_ascii_yyyyQq.zip, where “yyyy” represents the year and “q” the quarter). From 2012Q4 onward, the database was expanded and renamed FAERS (file names: faers_ascii_yyyyQq.zip), continuing through 2025Q2. This analysis focused on five primary files per quarter: DEMOyyQq.txt (patient demographics and administrative information), DRUGyyQq.txt (drug information), REACyyQq.txt (AE information), THERyyQq.txt (drug therapy start and end dates), and INDIyyQq.txt (indications for use), where “yy” indicates the final two digits of the year. To harmonize record formats between AERS and FAERS, discrepancies were resolved according to Food and Drug Administration documentation. FAERS employs a versioning system whereby each report update increases the {caseversion} without overwriting previous submissions; thus, only the most recent {caseversion} of each report was retained. For AERS data, where {caseversion} was unavailable, equivalent cases were identified using the unique identifiers {ISR} (unique number for identifying an AERS report) and {CASE} (number for identifying an AERS case). Variable names are presented in curly braces as designated in the database. Comprehensive preprocessing standardized the data for analysis. Event age {age} was uniformly converted to years. Inconsistent or anomalous entries in {sex}, {age}, and reporter country {reporter_country} were systematically reviewed and corrected. Weight data were available only for a small subset of reports and were not used in the analyses due to extensive missing data. Manual correction was required to address formatting errors in three AERS files, specifically restoring missing line breaks in DRUG11Q2.txt (line 322,967), DRUG11Q4.txt (line 446,738), and DRUG11Q3.txt (line 247,896). These corrections repaired file structures without altering the original report content. Through harmonization of AERS and FAERS records and rigorous quality control, a consistent and reliable dataset was established to support subsequent statistical analyses.

This study utilized de-identified, publicly available data from the FAERS database and did not involve human subjects research, direct patient interaction, or access to identifiable personal health information. Accordingly, institutional review board approval and informed consent were not required, in accordance with applicable institutional and national ethical guidelines.

Study design

This retrospective observational study analyzed four immunosuppressive regimens for SPK transplantation, as recorded in the FAERS database (2004Q1-2025Q2). The regimens were defined as follows: TMPB (tacrolimus, mycophenolate mofetil, prednisone, and basiliximab; basiliximab-containing regimen); TMP (tacrolimus, mycophenolate mofetil, and prednisone; triple-therapy regimen); TM (tacrolimus and mycophenolate mofetil; dual-therapy regimen); and T (tacrolimus monotherapy).

Inclusion criteria comprised reports indicating administration of these immunosuppressants for SPK transplantation during the study period. Immunosuppressant exposure was identified using the {drugname} variable (trade/brand names) for 2004Q1-2014Q2 data and primarily the {prod_ai} variable (active ingredients) from 2014Q3 onward; when {prod_ai} was missing, {drugname} was used. A comprehensive list of generic and brand names is provided in Appendix A. In this study, prednisone, prednisolone, and methylprednisolone were collectively treated as prednisone, and mycophenolate mofetil and mycophenolic acid were collectively treated as mycophenolate. Cases were classified as SPK transplantation when the {indi_pt} (Preferred Term-level medical terminology describing the Indication for use, using the Medical Dictionary for Regulatory Activities) variable indicated "renal and pancreas transplant" or both "pancreas transplant" and "renal transplant."

Exclusion criteria encompassed cases in which sirolimus, cyclosporine, everolimus, azathioprine, mizoribine, or rituximab were administered. To minimize heterogeneity and potential confounding from concomitant immunosuppressive agents, cases involving agents outside the predefined regimens were excluded. Additionally, duplicate entries-defined as reports sharing identical patient demographics, drug exposures, AE details, and relevant dates-were consolidated, with only one record retained for analysis.

AEs were categorized into transplant rejection, infections, renal disorders, pancreatic disorders, gastrointestinal disorders, hematological disorders, drug toxicity, and SAEs. Specific AEs included in each category are detailed in Appendix B. Transplant rejection served as the primary outcome of interest, with the other categories addressing safety outcomes.

Statistical analyses

Categorical data were presented as frequencies with reporting proportions (RPs) [7]. RP was calculated as (the number of SPK transplant recipients with the characteristic of interest) / (the total number of SPK transplant recipients reported in FAERS). Continuous variables were described using medians with first and third quartiles. Univariate logistic regression was used to estimate reporting odds ratios (RORs) [7] to assess associations between immunosuppressive regimens and AEs. Adjusted RORs (aRORs) were then obtained from multivariable binomial logistic regression models including {sex}, {age}, and {reporter_country} as covariates. Because of the high RP of missing data in FAERS, an available‑case approach was used when fitting the multivariable models: each candidate model was fitted using only cases with complete data on the variables included in that model, so sample sizes varied across models. Final models were selected by jointly considering sample size, statistical significance, and clinical plausibility. The {reporter_country} variable was recoded by continent, and the reference categories for regimen, sex, and continent were the TMPB group, female, and North America, respectively. Because the TMP, TM, and T groups were each compared with the TMPB group as the reference, yielding three primary comparisons, statistical significance was defined as p < 0.017 (=0.05/3) after Bonferroni correction. Correspondingly, 98.3% confidence intervals (CIs) were reported so that statistical significance aligned with 98.3% CIs excluding 1 [8]. Under this framework, RORs and aRORs retain the usual properties of odds ratios, and comparisons between regimen groups not directly tested (e.g., TM group versus T group) can be inferred from their reported estimates [9]. Associations were regarded as robust only when both unadjusted RORs (using all available reports) and aRORs (adjusted for background variables using available‑case data) met the *P* < 0.017 threshold, to reduce false positives arising from confounding or case-wise deletion. All analyses were conducted using R version 4.4.1 (R Foundation for Statistical Computing, Vienna, Austria). Data processing and statistical analyses followed the established FAERS analytical framework of previous study [7]. An explanation of the R code used in this study is provided in Appendix C. Because FAERS includes only reports with AEs and lacks data on unaffected patients, incidence rates could not be estimated. Accordingly, in line with previous pharmacovigilance studies [7], all results were expressed as disproportionality measures (RP, ROR, and aROR), which reflect patterns of spontaneous reporting rather than absolute risk. Because regimen assignment in FAERS may reflect post-transplant timing and clinical stability, this analysis was considered exploratory and was not intended to infer causal treatment effects.

To complement findings in SPK transplant recipients, the same analytical framework was applied to kidney‑only transplantation, allowing comparison of reporting trends between transplant populations.

## Results

Study population

**Figure 1:**
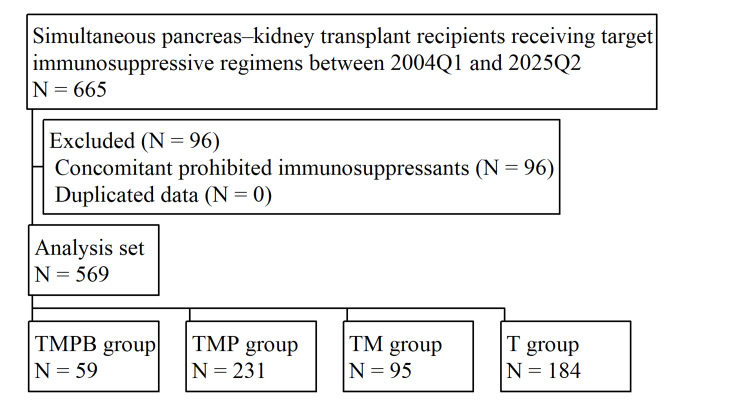
Flowchart of simultaneous pancreas-kidney transplant recipients receiving target immunosuppressive regimens. Image credit: All authors. TMPB, tacrolimus + mycophenolate mofetil + prednisone + basiliximab; TMP, tacrolimus + mycophenolate mofetil + prednisone; TM, tacrolimus + mycophenolate mofetil; T, tacrolimus monotherapy

From the FAERS database (2004Q1-2025Q2), 665 reports of SPK transplant recipients treated with the predefined immunosuppressive regimens were identified. After applying the exclusion criteria, 96 reports were excluded, leaving 569 reports for analysis. Figure 1 shows the distribution of reports across the four regimen groups: TMPB group (*N* = 59), TMP group (*N* = 231), TM group (*N* = 95), and T group (*N* = 184), and Table 1 summarizes their background characteristics.

**Table 1:** Summary of background information in simultaneous pancreas-kidney transplant recipients. Sex and country are summarized as frequencies (reporting proportions). Age is summarized as medians and first and third quartiles. Unknown for each variable is summarized as frequency (reporting proportion). Q1, first quartile; Q3, third quartile; TMPB, tacrolimus + mycophenolate mofetil + prednisone + basiliximab; TMP, tacrolimus + mycophenolate mofetil + prednisone; TM, tacrolimus + mycophenolate mofetil; T, tacrolimus monotherapy

Characteristics	TMPB group (*N* = 59)	TMP group (*N* = 231)	TM group (*N* = 95)	T group (*N* = 184)
Sex, *n* (%)				
Female	20 (33.9)	87 (37.7)	37 (38.9)	75 (40.8)
Male	23 (39.0)	110 (47.6)	49 (51.6)	92 (50.0)
Unknown	16 (27.1)	34 (14.7)	9 (9.5)	17 (9.2)
Age (years)				
Median	43.0	45.0	45.0	46.0
Q1-Q3	37.0-48.0	38.0-52.0	40.0-52.0	37.5-54.0
Unknown, *n* (%)	18 (30.5)	58 (25.1)	17 (17.9)	61 (33.2)
Continent, *n* (%)				
North America	9 (15.3)	131 (56.7)	55 (57.9)	141 (76.6)
South America	1 (1.7)	6 (2.6)	4 (4.2)	8 (4.3)
Asia	17 (28.8)	9 (3.9)	3 (3.2)	8 (4.3)
Europe	30 (50.8)	72 (31.2)	31 (32.6)	12 (6.5)
Africa	0 (0.0)	0 (0.0)	1 (1.1)	0 (0.0)
Oceania	2 (3.4)	5 (2.2)	0 (0.0)	2 (1.1)
Unknown	0 (0.0)	8 (3.5)	1 (1.1)	13 (7.1)

Across all groups, males outnumbered females, with unknown sex reported in 9.2-27.1% of cases. Median age was similar among groups, ranging from 43 to 46 years, although age was missing in 17.9-33.2% of reports. In terms of geography, most reports in the TMP, TM, and T groups were from North America, whereas the TMPB group included a higher proportion of reports from Europe and Asia.

Adverse events

Table 2 summarizes the distribution of AE categories across regimens, and Table 3 presents the corresponding RORs and aRORs (with the TMPB group as the reference).

**Table 2:** Summary of adverse event categories in simultaneous pancreas-kidney transplant recipients. Data are summarized as frequencies (reporting proportions). TMPB, tacrolimus + mycophenolate mofetil + prednisone + basiliximab; TMP, tacrolimus + mycophenolate mofetil + prednisone; TM, tacrolimus + mycophenolate mofetil; T, tacrolimus monotherapy

Adverse event category	TMPB group (N = 59)	TMP group (N = 231)	TM group (N = 95)	T group (N = 184)
Transplant rejection	28 (47.5)	57 (24.7)	11 (11.6)	23 (12.5)
Infections	17 (28.8)	59 (25.5)	12 (12.6)	18 (9.8)
Renal disorders	6 (10.2)	32 (13.9)	13 (13.7)	25 (13.6)
Pancreatic disorders	4 (6.8)	15 (6.5)	4 (4.2)	7 (3.8)
Gastrointestinal disorders	3 (5.1)	23 (10.0)	10 (10.5)	19 (10.3)
Hematological disorders	2 (3.4)	22 (9.5)	8 (8.4)	13 (7.1)
Drug toxicity	3 (5.1)	15 (6.5)	9 (9.5)	6 (3.3)
Serious adverse events	6 (10.2)	21 (9.1)	9 (9.5)	35 (19.0)

**Table 3:** The ROR and aROR for adverse event categories in simultaneous pancreas-kidney transplant recipients. For RORs and aRORs, the reference categories for regimen, sex, and continent were the TMPB group, female sex, and North America, respectively. Statistical significance was defined as *P* < 0.017 after Bonferroni correction for multiple comparisons. Sample size used for ROR estimation was 569 for regimen, 493 for sex, 415 for age, and 547 for continent. Sample size used for aROR estimation was 547 for transplant rejection, 493 for infections, renal disorders, and hematological disorders, 411 for pancreatic disorders, and 569 for gastrointestinal disorders, drug toxicity, and serious adverse events. †: not calculated due to zero cell count. -: not selected in multivariate analysis. aROR, adjusted reporting odds ratio; CI, confidence interval; ROR, reporting odds ratio; TMPB, tacrolimus + mycophenolate mofetil + prednisone + basiliximab; TMP, tacrolimus + mycophenolate mofetil + prednisone; TM, tacrolimus + mycophenolate mofetil; T, tacrolimus monotherapy

Adverse event category	Univariate analysis		Multivariate analysis	
	ROR (98.3% CI)	*P*-value	aROR (98.3% CI)	*P*-value
Transplant rejection				
TMP group	0.363 (0.176-0.748)	0.001	0.400 (0.177-0.906)	0.007
TM group	0.145 (0.054-0.390)	<0.001	0.151 (0.052-0.441)	<0.001
T group	0.158 (0.070-0.360)	<0.001	0.236 (0.090-0.616)	<0.001
Male	0.742 (0.414-1.329)	0.221	-	-
Age	0.979 (0.951-1.008)	0.087	-	-
South America	6.300 (1.958-20.272)	<0.001	6.870 (2.066-22.848)	<0.001
Asia	1.931 (0.686-5.433)	0.128	1.005 (0.314-3.215)	0.991
Europe	4.154 (2.349-7.346)	<0.001	3.144 (1.679-5.886)	<0.001
Africa	†	†	†	†
Oceania	2.000 (0.282-14.194)	0.397	1.282 (0.170-9.666)	0.769
Infections				
TMP group	0.847 (0.390-1.844)	0.610	0.561 (0.237-1.325)	0.107
TM group	0.357 (0.130-0.981)	0.015	0.243 (0.083-0.712)	0.002
T group	0.268 (0.108-0.665)	0.001	0.155 (0.057-0.419)	<0.001
Male	0.427 (0.245-0.742)	<0.001	0.406 (0.229-0.720)	<0.001
Age	1.009 (0.983-1.036)	0.393	-	-
South America	2.252 (0.611-8.293)	0.136	-	-
Asia	4.298 (1.770-10.437)	<0.001	-	-
Europe	1.858 (1.012-3.411)	0.015	-	-
Africa	†	†	-	-
Oceania	1.801 (0.255-12.745)	0.472	-	-
Renal disorders				
TMP group	1.420 (0.460-4.386)	0.456	1.132 (0.352-3.635)	0.800
TM group	1.400 (0.399-4.910)	0.520	1.029 (0.280-3.787)	0.958
T group	1.389 (0.439-4.398)	0.495	0.704 (0.206-2.407)	0.494
Male	0.449 (0.234-0.864)	0.003	0.446 (0.232-0.860)	0.003
Age	0.985 (0.954-1.017)	0.248	-	-
South America	5.703 (1.741-18.679)	<0.001	-	-
Asia	1.225 (0.361-4.162)	0.691	-	-
Europe	1.328 (0.660-2.673)	0.331	-	-
Africa	†	†	-	-
Oceania	2.241 (0.315-15.959)	0.325	-	-
Pancreatic disorders				
TMP group	0.955 (0.237-3.852)	0.937	3.278 (0.253-42.550)	0.267
TM group	0.604 (0.106-3.449)	0.489	3.084 (0.195-48.684)	0.329
T group	0.544 (0.116-2.550)	0.345	2.708 (0.194-37.702)	0.365
Male	0.261 (0.089-0.764)	0.003	0.258 (0.079-0.843)	0.006
Age	0.921 (0.872-0.972)	<0.001	0.927 (0.879-0.978)	0.001
South America	3.476 (0.502-24.050)	0.123	-	-
Asia	3.581 (0.828-15.492)	0.037	-	-
Europe	2.666 (0.952-7.461)	0.023	-	-
Africa	†	†	-	-
Oceania	†	†	-	-
Gastrointestinal disorders				
TMP group	2.064 (0.455-9.372)	0.252	2.064 (0.455-9.372)	0.252
TM group	2.196 (0.431-11.195)	0.248	2.196 (0.431-11.195)	0.248
T group	2.149 (0.464-9.953)	0.232	2.149 (0.464-9.953)	0.232
Male	0.823 (0.383-1.769)	0.542	-	-
Age	0.995 (0.959-1.032)	0.739	-	-
South America	6.955 (2.015-24.011)	<0.001	-	-
Asia	1.863 (0.533-6.507)	0.234	-	-
Europe	1.479 (0.664-3.294)	0.242	-	-
Africa	†	†	-	-
Oceania	†	†	-	-
Hematological disorders				
TMP group	3.000 (0.494-18.220)	0.145	2.427 (0.386-15.260)	0.248
TM group	2.621 (0.378-18.165)	0.234	2.227 (0.312-15.908)	0.330
T group	2.167 (0.339-13.847)	0.318	0.509 (0.061-4.272)	0.447
Male	0.308 (0.122-0.779)	0.002	0.296 (0.116-0.757)	0.002
Age	0.975 (0.935-1.017)	0.146	-	-
South America	9.732 (2.730-34.699)	<0.001	-	-
Asia	0.953 (0.153-5.944)	0.950	-	-
Europe	1.505 (0.602-3.765)	0.285	-	-
Africa	†	†	-	-
Oceania	2.086 (0.155-28.118)	0.499	-	-
Drug toxicity				
TMP group	1.296 (0.273-6.145)	0.690	1.296 (0.273-6.145)	0.690
TM group	1.953 (0.376-10.152)	0.331	1.953 (0.376-10.152)	0.331
T group	0.629 (0.111-3.557)	0.522	0.629 (0.111-3.557)	0.522
Male	1.489 (0.590-3.759)	0.303	-	-
Age	1.022 (0.981-1.065)	0.200	-	-
South America	†	†	-	-
Asia	1.143 (0.181-7.235)	0.862	-	-
Europe	2.308 (0.942-5.652)	0.025	-	-
Africa	†	†	-	-
Oceania	†	†	-	-
Serious adverse events				
TMP group	0.883 (0.275-2.840)	0.799	0.883 (0.275-2.840)	0.799
TM group	0.924 (0.245-3.492)	0.887	0.924 (0.245-3.492)	0.887
T group	2.075 (0.674-6.391)	0.120	2.075 (0.674-6.391)	0.120
Male	1.099 (0.562-2.152)	0.736	-	-
Age	0.977 (0.945-1.010)	0.098	-	-
South America	4.861 (1.443-16.373)	0.002	-	-
Asia	0.735 (0.164-3.303)	0.624	-	-
Europe	1.333 (0.652-2.725)	0.335	-	-
Africa	†	†	-	-
Oceania	1.042 (0.079-13.666)	0.970	-	-

Transplant rejection was most frequent in the TMPB group (47.5%) and showed lower RPs in the TMP group (24.7%), TM group (11.6%), and T group (12.5%). Using the Bonferroni-corrected threshold (*P* < 0.017), both RORs and aRORs were significantly lower in the TMP group (ROR: 0.363, 98.3% CI: 0.176-0.748, *P* = 0.001; aROR: 0.400, 98.3% CI: 0.177-0.906, *P* = 0.007), TM group (ROR: 0.145, 98.3% CI: 0.054-0.390, *P* < 0.001; aROR: 0.151, 98.3% CI: 0.052-0.441, *P* < 0.001), and T group (ROR: 0.158, 98.3% CI: 0.070-0.360, *P* < 0.001; aROR: 0.236, 98.3% CI: 0.090-0.616, *P* < 0.001).

Infections were more frequently reported in the TMPB group (28.8%) and TMP group (25.5%) and less frequently reported in the TM group (12.6%) and T group (9.8%). Relative to the TMPB group, RORs for infections were significantly lower in the TM and T groups, and these reductions remained significant for the TM group (ROR: 0.357, 98.3% CI: 0.130-0.981, *P* = 0.015; aROR 0.243, 98.3% CI: 0.083-0.712, *P* = 0.002) and T group (ROR: 0.268, 98.3% CI: 0.108-0.665, *P* = 0.001; aROR 0.155, 98.3% CI: 0.057-0.419, *P* < 0.001). For renal, pancreatic, gastrointestinal, hematological disorders, and drug toxicity, RPs were broadly similar across regimens, and neither RORs nor aRORs for the TMP group, TM group, or T group versus the TMPB group reached the Bonferroni‑corrected significance level. For SAEs, the RP was higher in the T group (19.0%) than in the TMPB group (10.2%), TMP group (9.1%), and TM group (9.5%). However, neither the ROR nor the aROR reached statistical significance at *P* < 0.017. Thus, while SAE reporting was numerically higher in the T group, the association did not reach the pre-specified Bonferroni-adjusted significance level and should be interpreted cautiously as a potential reporting trend rather than a demonstrated regimen effect.

To explore the possibility that the observed between‑regimen differences may partly reflect geographic reporting patterns, we performed continent‑stratified analyses for regions with ≥30 SPK recipients, the results of which are summarized in Appendix D. Across the continents for which these analyses were feasible, the trends of lower rejection and infection reporting in the TM and T groups were broadly consistent with the overall findings, although some variation in reporting ratios for individual AE categories was observed.

Kidney-only transplantation

The same statistical framework was applied to kidney‑only transplantation. Corresponding figure and tables for kidney‑only transplant recipients are shown in Appendices E-H. A total of 16,892 reports involving the targeted immunosuppressive regimens were identified from the FAERS database (2004Q1-2025Q2). These reports were distributed among the four groups as follows: TMPB group (*N* = 1,040), TMP group (*N* = 6,033), TM group (*N* = 3,917), and T group (*N* = 5,902). Background characteristics, including sex and continent distributions, were generally similar to those observed in SPK transplant recipients. However, kidney‑only transplant recipients tended to be older, with median ages approximately five to nine years higher across all groups.

Reporting patterns showed trends similar to those in SPK transplantation. Transplant rejection occurred most frequently in the TMPB group (19.9%), although at lower RPs than those observed in SPK transplant recipients. The RP of transplant rejection progressively declined in the TMP group (9.1%), TM group (6.1%), and T group (4.1%). Infections showed a similar decreasing trend, ranging from 21.8% in the TMPB group to 7.0% in the T group. Renal disorders were more prevalent among reports in the TMPB and TMP groups than in the TM and T groups. By contrast, SAEs were lowest in the TMPB group but progressively increased in the TMP, TM, and T groups. The larger sample size yielded more statistically significant RORs and aRORs (*P* < 0.017), although the overall reporting patterns were qualitatively consistent across transplant types.

Corresponding continent-stratified analyses for kidney-only transplant recipients are presented in Appendix I and showed qualitatively similar patterns.

## Discussion

This pharmacovigilance study evaluated AE reporting among SPK transplant recipients receiving four commonly used immunosuppressive regimens in routine clinical practice. The lower reporting of rejection and infection in the TM and T groups likely reflects the later post-transplant phase, clinical stability, and survivor-related selection rather than a direct regimen effect, because FAERS does not capture transplant timing or allow adequate adjustment for time-related confounding. Accordingly, these differences should be interpreted as hypothesis-generating reporting signals rather than causal effects of specific tapering strategies. The higher infection reporting in the TMPB and TMP groups is consistent with early post-transplant vulnerability, whereas the T group showed numerically higher SAE reporting, although this was not statistically significant. Overall, these findings suggest that immunosuppressive de-escalation after SPK transplantation warrants cautious interpretation in the context of post-transplant timing and requires prospective validation.

Tacrolimus monotherapy has been proposed as a strategy to simplify post‑transplant care, improve adherence, and reduce pill burden, particularly in immunologically low‑risk recipients [10,11]. However, concerns persist that reliance on a single calcineurin inhibitor may exacerbate chronic nephrotoxicity and disturb immune homeostasis, given the well‑recognized chronic toxicities of calcineurin inhibitors. In contrast, combination therapy with tacrolimus and mycophenolate mofetil provides complementary mechanisms of action that may allow for lower tacrolimus exposure while maintaining adequate immunosuppression [10]. Mycophenolate mofetil, however, is associated with gastrointestinal and hematologic toxicity [12] and imposes additional economic burden for patients and healthcare systems, which may limit its long-term use in some settings. Within this context, the T group showed numerically higher SAE reporting, but this non-significant observation should be interpreted cautiously and not as evidence of inferior safety.

In this study, male recipients consistently outnumbered female recipients across all groups, mirroring previously reported patterns in SPK transplantation [2,12]. This sex imbalance may reflect a combination of factors, including the higher prevalence of diabetes and end-stage renal disease in males, sex-related immunologic differences that may complicate transplant candidacy in females, and anatomical considerations such as graft-recipient size mismatch, which can disadvantage smaller female candidates [12]. The median age of SPK transplant recipients was five to nine years younger than that of kidney-only recipients [2], consistent with differences in disease etiology, referral practices, and center-level selection practices that may favor younger candidates for SPK to maximize long-term survival benefit [13,14]. The higher frequency of transplant rejection in SPK recipients compared with kidney-only recipients observed in this analysis is also in line with previous literature [3,15] and likely reflects the additional immunologic challenge posed by pancreatic tissue, particularly the vulnerability of islet cells to immune-mediated injury.

Management of dual-organ transplant recipients introduces additional complexity to immunosuppressive decision-making and long-term follow-up. Compared with kidney-only recipients, SPK recipients may have higher rejection rates, possibly reflecting the additional immunogenic burden of pancreatic tissue and the greater immunologic complexity of dual-organ transplantation [3]. These observations underscore the need for SPK-specific immunomodulatory strategies rather than simple extrapolation from kidney-only transplantation data. Achieving an optimal balance between sufficient immunosuppression to protect both grafts and minimization of long-term toxicity remains a central challenge in this population, and our data indicate that regimen de-escalation should be interpreted in the context of the post-transplant phase and clinical stability, with the observed SAE pattern in the T group remaining non-significant and exploratory.

This study also has several notable strengths. It leveraged a large, internationally sourced pharmacovigilance database spanning more than two decades, enabling evaluation of relatively rare procedures such as SPK transplantation across diverse healthcare settings. By focusing on clearly defined immunosuppressive regimens and applying consistent exclusion criteria, the analysis reduced heterogeneity from concomitant agents and facilitated the supporting comparison of stepwise de‑escalation strategies. In addition, parallel analyses in kidney‑only transplant recipients provided an external reference, allowing assessment of whether observed reporting patterns were specific to SPK or reflected broader trends in solid organ transplantation.

Several limitations must also be acknowledged. First, the pharmacovigilance database captures only cases with reported AEs and does not include patients without AEs, precluding estimation of true incidence rates or absolute risks for each regimen. Second, spontaneous reporting is subject to underreporting and various forms of reporting bias, with severe, unusual, or suspected drug‑related events more likely to be reported than mild or expected events, which may distort apparent associations between regimens and AEs. Third, key clinical data, including medication adherence, comorbidities, and detailed transplant‑related information, were often incomplete or absent, limiting adjustment for confounding by indication and center‑specific practices. Fourth, the cross-sectional structure and drug-focused design of the database hinder reconstruction of precise timelines, so the four regimens (TMPB, TMP, TM, and T) had to be inferred from reported drug combinations, introducing potential misclassification. Because FAERS does not reliably capture time since transplantation, time- and indication-related confounding could not be adequately addressed. TMPB is generally used early after transplantation when rejection and infection risks are highest, whereas TM and T regimens are typically administered years later in clinically stable patients. Therefore, regimen comparisons in FAERS should not be interpreted as causal treatment effects. As a result, the observed differences in AE reporting between regimens may partly reflect underlying differences in post‑transplant time and survivor bias rather than intrinsic regimen effects. Consequently, our findings should be interpreted as hypothesis-generating with respect to stepwise de-escalation strategies rather than as definitive comparative safety estimates. Finally, unequal sample sizes across regimens and the available‑case approach to missing data may have reduced statistical power and introduced selection bias, and therefore, the present findings should be interpreted as hypothesis‑generating rather than definitive evidence of comparative safety or effectiveness. Weight data were missing for most reports and were excluded from the analyses, which also limited baseline characterization. Sensitivity analyses by continent showed that the main regimen‑specific patterns, such as lower rejection and infection reporting in the TM and T groups, were broadly consistent across continents for which these analyses were feasible, suggesting that the observed differences between regimens are unlikely to be fully explained by geographic reporting heterogeneity alone. That said, the limited sample size in some regions precludes strong conclusions regarding the contribution of regional factors to the overall findings.

Despite these caveats, the findings may provide clinically relevant signals regarding post-transplant immunosuppressive strategies in SPK recipients. Intensive multidrug immunosuppression is indispensable in the early postoperative period to prevent acute rejection and early graft loss, but progressive tapering is essential to reduce cumulative toxicity and improve long‑term tolerability. The present analysis showed a lower reporting pattern of transplant rejection and infections in the TM group. The T group showed numerically higher SAE reporting, but this finding was not statistically significant and should be regarded as a signal requiring confirmation in prospective studies. Collectively, these findings indicate distinct reporting patterns across regimens and underscore the need for prospective validation in SPK recipients as well as vigilant monitoring for infections and other AEs, particularly during the tacrolimus monotherapy phase. Future prospective and population‑based studies, ideally incorporating detailed clinical, laboratory, and pharmacokinetic data, are required to validate these observations and to refine immunosuppressive strategies that optimize long‑term outcomes for this complex and vulnerable patient population.

## Conclusions

In this exploratory pharmacovigilance analysis of SPK transplant recipients, lower reporting of transplant rejection and infections was observed in the TM group. However, because regimen assignment likely reflects post-transplant phase and clinical stability, the observed differences should be interpreted as hypothesis-generating reporting patterns rather than evidence of comparative safety. Given the inherent limitations of spontaneous reporting data, these findings should be regarded as hypothesis‑generating and confirmed in future prospective and population‑based studies.

## Figures and Tables

**Figure 2:**
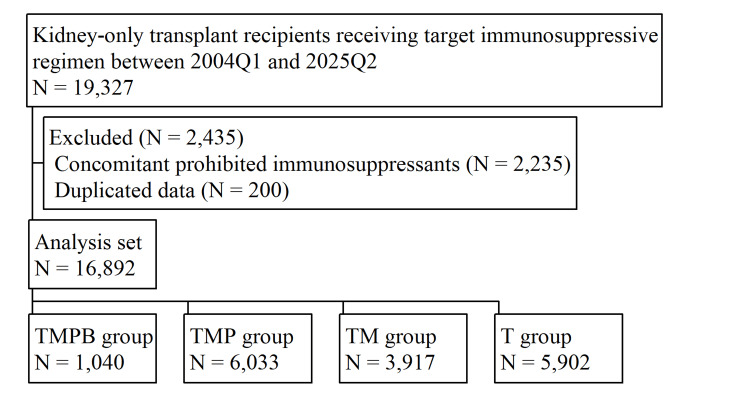
Flowchart of kidney-only transplant recipients receiving target immunosuppressive regimens TMPB, tacrolimus + mycophenolate mofetil + prednisone + basiliximab; TMP, tacrolimus + mycophenolate mofetil + prednisone; TM, tacrolimus + mycophenolate mofetil; T, tacrolimus monotherapy

## Appendices

Appendix A

**Table 4:** Generic and brand names of immunosuppressants Generic and brand names are presented exactly as they appear in the United States Food and Drug Administration Adverse Event Reporting System to maintain consistency with the original database and to ensure searchability

Study drug status	Generic name	Brand name
Included drug	Tacrolimus	Adoport, Advagraf, Astagraf, Envarsus, Graceptor, Prograf, Tacsant
	Mycophenolate mofetil, Mycophenolic acid	Cellcept, Myfortic
	Prednisone, Prednisolone, Methylprednisolone	Aprednislon, Cortancyl, Decortin, Medrol, Prednisolon, Prednison, Prednisona, Predonine, Solupred, Urbason
	Basiliximab	Simulect
Excluded drug	Sirolimus	Fyarro, Hyftor, Rapamune
	Cyclosporine	Ciclosporin, Ciclosporine, Cyclosporin, Equoral, Gengraf, Neoral, Sandimmune
	Everolimus	Afinitor, Certican, Rad001, Sdz-rad, Zortress
	Azathioprine	Azathioprin, Azanin, Azasan, Imuran, Imurek, Imurel, Zytrim
	Mizoribine	Bredinin
	Rituximab	Mabthera, Rituxan

Appendix B

**Table 5:** Categorization of preferred terms Preferred terms are presented exactly as they appear in the United States Food and Drug Administration Adverse Event Reporting System to maintain consistency with the original database and to ensure searchability

Adverse event category	Preferred terms
Transplant rejection	Kidney transplant rejection, Pancreas transplant rejection, Renal and pancreas transplant rejection, Transplant rejection
Infections	Bronchopulmonary aspergillosis, COVID-19, Cytomegalovirus infection, Cytomegalovirus viraemia, Endocarditis, Epstein-Barr virus infection, Herpes zoster, Infection, Pneumonia, Pseudomonas infection, Sepsis, Staphylococcal infection, Urinary tract infection
Renal disorders	Acute kidney injury, Blood creatinine increased, Blood urea increased, Chronic kidney disease, Haemodialysis, Nephropathy toxic, Polyomavirus-associated nephropathy, Proteinuria, Pyelonephritis, Renal disorder, Renal failure, Renal failure acute, Renal failure chronic, Renal impairment
Pancreatic disorders	Complications of transplanted pancreas, Pancreatic leak, Pancreatic necrosis, Pancreatic pseudoaneurysm, Pancreatic pseudocyst, Pancreatitis
Gastrointestinal disorders	Abdominal discomfort, Abdominal pain, Diarrhoea, Gastrointestinal disorder, Gastrointestinal haemorrhage, Nausea, Vomiting
Hematological disorders	Anaemia, Blood lactate dehydrogenase increased, Haematocrit decreased, Haemoglobin decreased, Haemolytic uraemic syndrome, Leukopenia, Neutropenia, Pancytopenia, Thrombocytopenia, Thrombotic microangiopathy, White blood cell count decreased
Drug toxicity	Toxicity to various agents, Drug interaction
Serious adverse events	Cerebrovascular accident, Death, Graft loss, Hospitalisation, Hypoxia, Respiratory failure, Shock haemorrhagic

Appendix C

The R code used in this study was based on the scripts provided in Supplementary material G of previous study [7]. The following key modifications were introduced to adapt the analysis to simultaneous pancreas-kidney transplantation: 1. Changed the FAERS data extraction period to 2004Q1-2025Q2 (end_y = 2025, end_q = 2). 2. Redefined drug.name1-drug.name10 according to the target and prohibited immunosuppressants listed in Appendix A. 3. Changed the extraction method to include cases where the {indi_pt} variable indicates "renal and pancreas transplant" or both "pancreas transplant" and "renal transplant " within the same caseid. 4. Re-grouped preferred terms (pt1-pt8) into clinically meaningful AE categories aligned with Appendix B. 5. Restricted the analysis to four regimen patterns ("1111000000", "1110000000", "1100000000", "1000000000") across drug.name1-drug.name10 (1 = administered, 0 = not administered), corresponding to the TMPB, TMP, TM, and T groups.

Appendix D

**Table 6:** Summary of adverse event categories in simultaneous pancreas-kidney transplant recipients by continent Data are summarized as frequencies (reporting proportions). TMPB, tacrolimus + mycophenolate mofetil + prednisone + basiliximab; TMP, tacrolimus + mycophenolate mofetil + prednisone; TM, tacrolimus + mycophenolate mofetil; T, tacrolimus monotherapy

Continent	Adverse event category	TMPB group	TMP group	TM group	T group
North America		N = 59	N = 231	N = 95	N = 184
	Transplant rejection	1 (11.1)	23 (17.6)	3 (5.5)	15 (10.6)
	Infections	2 (22.2)	27 (20.6)	7 (12.7)	10 (7.1)
	Renal disorders	0 (0.0)	15 (11.5)	9 (16.4)	14 (9.9)
	Pancreatic disorders	2 (22.2)	5 (3.8)	1 (1.8)	3 (2.1)
	Gastrointestinal disorders	1 (11.1)	9 (6.9)	6 (10.9)	10 (7.1)
	Hematological disorders	0 (0.0)	10 (7.6)	5 (9.1)	4 (2.8)
	Drug toxicity	0 (0.0)	7 (5.3)	6 (10.9)	3 (2.1)
	Serious adverse events	0 (0.0)	4 (3.1)	6 (10.9)	26 (18.4)
Asia		N = 17	N = 9	N = 3	N = 8
	Transplant rejection	5 (29.4)	2 (22.2)	1 (33.3)	0 (0.0)
	Infections	10 (58.8)	3 (33.3)	1 (33.3)	1 (12.5)
	Renal disorders	3 (17.6)	0 (0.0)	0 (0.0)	2 (25.0)
	Pancreatic disorders	2 (11.8)	0 (0.0)	0 (0.0)	2 (25.0)
	Gastrointestinal disorders	1 (5.9)	3 (33.3)	0 (0.0)	1 (12.5)
	Hematological disorders	1 (5.9)	0 (0.0)	0 (0.0)	1 (12.5)
	Drug toxicity	0 (0.0)	1 (11.1)	1 (33.3)	0 (0.0)
	Serious adverse events	0 (0.0)	1 (11.1)	1 (33.3)	1 (12.5)
Europe		N = 30	N = 72	N = 31	N = 12
	Transplant rejection	21 (70.0)	28 (38.9)	5 (16.1)	0 (0.0)
	Infections	4 (13.3)	23 (31.9)	4 (12.9)	2 (16.7)
	Renal disorders	3 (10.0)	14 (19.4)	3 (9.7)	1 (8.3)
	Pancreatic disorders	0 (0.0)	8 (11.1)	3 (9.7)	1 (8.3)
	Gastrointestinal disorders	1 (3.3)	10 (13.9)	4 (12.9)	1 (8.3)
	Hematological disorders	1 (3.3)	8 (11.1)	3 (9.7)	0 (0.0)
	Drug toxicity	3 (10.0)	7 (9.7)	2 (6.5)	3 (25.0)
	Serious adverse events	5 (16.7)	13 (18.1)	1 (3.2)	1 (8.3)

Appendix E

Appendix F

**Table 7:** Summary of background information in kidney-only transplant recipients Sex and country are summarized as frequencies (reporting proportions). Age is summarized as medians and first and third quartiles. Unknown for each variable is summarized as frequency (reporting proportion). Q1, first quartile; Q3, third quartile; TMPB, tacrolimus + mycophenolate mofetil + prednisone + basiliximab; TMP, tacrolimus + mycophenolate mofetil + prednisone; TM, tacrolimus + mycophenolate mofetil; T, tacrolimus monotherapy

Characteristics	TMPB group	TMP group	TM group	T group
	N = 1,040	N = 6,033	N = 3,917	N = 5,902
Sex, n (%)				
Female	369 (35.5)	2,216 (36.7)	1,473 (37.6)	2,327 (39.4)
Male	612 (58.8)	3,346 (55.5)	2,172 (55.5)	3,203 (54.3)
Unknown	59 (5.7)	471 (7.8)	272 (6.9)	372 (6.3)
Age, years				
Median	48.0	53.0	54.0	55.0
Q1–Q3	34.0-58.5	38.0-63.0	41.0-65.0	41.0-64.0
Unknown, n (%)	101 (9.7)	896 (14.9)	1,083 (27.6)	1,674 (28.4)
Continent, n (%)				
North America	194 (18.7)	2,230 (37.0)	1,922 (49.1)	3,302 (55.9)
South America	18 (1.7)	386 (6.4)	173 (4.4)	594 (10.1)
Asia	354 (34.0)	913 (15.1)	581 (14.8)	736 (12.5)
Europe	394 (37.9)	2,298 (38.1)	1,093 (27.9)	995 (16.9)
Africa	5 (0.5)	30 (0.5)	32 (0.8)	20 (0.3)
Oceania	41 (3.9)	67 (1.1)	13 (0.3)	42 (0.7)
Unknown	34 (3.3)	109 (1.8)	103 (2.6)	213 (3.6)

Appendix G

**Table 8:** Summary of adverse event categories in kidney-only transplant recipients Data are summarized as frequencies (reporting proportions). TMPB, tacrolimus + mycophenolate mofetil + prednisone + basiliximab; TMP, tacrolimus + mycophenolate mofetil + prednisone; TM, tacrolimus + mycophenolate mofetil; T, tacrolimus monotherapy

Adverse event category	TMPB group	TMP group	TM group	T group
	N = 1,040	N = 6,033	N = 3,917	N = 5,902
Transplant rejection	207 (19.9)	552 (9.1)	238 (6.1)	243 (4.1)
Infections	227 (21.8)	1,125 (18.6)	546 (13.9)	414 (7.0)
Renal disorders	290 (27.9)	1,056 (17.5)	500 (12.8)	452 (7.7)
Pancreatic disorders	7 (0.7)	12 (0.2)	2 (0.1)	14 (0.2)
Gastrointestinal disorders	133 (12.8)	529 (8.8)	338 (8.6)	325 (5.5)
Hematological disorders	216 (20.8)	558 (9.2)	255 (6.5)	133 (2.3)
Drug toxicity	76 (7.3)	483 (8.0)	213 (5.4)	311 (5.3)
Serious adverse events	36 (3.5)	356 (5.9)	447 (11.4)	728 (12.3)

Appendix H

**Table 9:** The ROR and aROR for adverse event categories in kidney-only transplant recipients For RORs and aRORs, the reference categories for regimen, sex, and continent were the TMPB group, female sex, and North America, respectively. Statistical significance was defined as p < 0.017 after Bonferroni correction for multiple comparisons. Sample size used for ROR estimation was 16,892 for regimen, 15,718 for sex, 13,138 for age, and 16,433 for continent. Sample size used for aROR estimation was 12,770 for transplant rejection, 13,138 for infections, 15,293 for renal disorders, hematological disorders, and drug toxicity, 16,892 for pancreatic disorders, 15,718 for gastrointestinal disorders, 12,566 for serious adverse events. †: not calculated due to zero cell count. -: not selected in multivariate analysis. aROR, adjusted reporting odds ratio; CI, confidence interval; ROR, reporting odds ratio; TMPB, tacrolimus + mycophenolate mofetil + prednisone + basiliximab; TMP, tacrolimus + mycophenolate mofetil + prednisone; TM, tacrolimus + mycophenolate mofetil; T, tacrolimus monotherapy

Adverse event category	Univariate analysis		Multivariate analysis	
	ROR (98.3% CI)	p-value	aROR (98.3% CI)	p-value
Transplant rejection				
TMP group	0.405 (0.327–0.502)	<0.001	0.411 (0.321–0.525)	<0.001
TM group	0.260 (0.204–0.333)	<0.001	0.281 (0.209–0.378)	<0.001
T group	0.173 (0.135–0.220)	<0.001	0.185 (0.137–0.249)	<0.001
Male	0.940 (0.806–1.097)	0.337	-	-
Age	0.973 (0.968–0.977)	<0.001	0.973 (0.969–0.978)	<0.001
South America	2.388 (1.878–3.038)	<0.001	2.190 (1.667–2.878)	<0.001
Asia	1.677 (1.373–2.049)	<0.001	0.978 (0.764–1.251)	0.826
Europe	1.334 (1.119–1.591)	<0.001	0.900 (0.728–1.112)	0.233
Africa	0.999 (0.330–3.028)	0.998	0.265 (0.047–1.501)	0.067
Oceania	2.691 (1.551–4.671)	<0.001	1.868 (1.009–3.458)	0.015
Infections				
TMP group	0.821 (0.675–0.999)	0.016	0.840 (0.683–1.034)	0.045
TM group	0.580 (0.470–0.716)	<0.001	0.545 (0.433–0.687)	<0.001
T group	0.270 (0.217–0.336)	<0.001	0.267 (0.210–0.338)	<0.001
Male	0.936 (0.837–1.046)	0.154	-	-
Age	1.004 (1.001–1.008)	0.005	1.006 (1.003–1.010)	<0.001
South America	1.822 (1.499–2.214)	<0.001	-	-
Asia	1.270 (1.085–1.488)	<0.001	-	-
Europe	1.285 (1.129–1.462)	<0.001	-	-
Africa	1.073 (0.493–2.334)	0.828	-	-
Oceania	1.954 (1.222–3.124)	0.001	-	-
Renal disorders				
TMP group	0.549 (0.456–0.660)	<0.001	0.572 (0.470–0.697)	<0.001
TM group	0.378 (0.309–0.463)	<0.001	0.414 (0.333–0.513)	<0.001
T group	0.214 (0.175–0.263)	<0.001	0.238 (0.191–0.297)	<0.001
Male	1.188 (1.059–1.333)	<0.001	1.131 (1.004–1.274)	0.014
Age	0.997 (0.994–1.001)	0.073	-	-
South America	2.163 (1.758–2.661)	<0.001	2.395 (1.933–2.968)	<0.001
Asia	2.023 (1.726–2.370)	<0.001	1.647 (1.389–1.953)	<0.001
Europe	2.302 (2.020–2.624)	<0.001	1.876 (1.628–2.161)	<0.001
Africa	1.169 (0.501–2.730)	0.659	0.987 (0.400–2.437)	0.973
Oceania	2.102 (1.267–3.487)	<0.001	1.508 (0.888–2.559)	0.063
Pancreatic disorders				
TMP group	0.294 (0.094–0.921)	0.010	0.294 (0.094–0.921)	0.010
TM group	0.075 (0.011–0.515)	0.001	0.075 (0.011–0.515)	0.001
T group	0.351 (0.116–1.066)	0.024	0.351 (0.116–1.066)	0.024
Male	0.947 (0.396–2.266)	0.882	-	-
Age	1.011 (0.985–1.038)	0.322	-	-
South America	0.871 (0.143–5.286)	0.854	-	-
Asia	0.987 (0.286–3.400)	0.979	-	-
Europe	1.388 (0.560–3.441)	0.388	-	-
Africa	†	†	-	-
Oceania	†	†	-	-
Gastrointestinal disorders				
TMP group	0.655 (0.512–0.840)	<0.001	0.675 (0.523–0.872)	<0.001
TM group	0.644 (0.496–0.836)	<0.001	0.661 (0.506–0.865)	<0.001
T group	0.397 (0.306–0.516)	<0.001	0.412 (0.315–0.539)	<0.001
Male	0.737 (0.640–0.848)	<0.001	0.726 (0.630–0.835)	<0.001
Age	1.000 (0.995–1.004)	0.805	-	-
South America	2.269 (1.817–2.835)	<0.001	-	-
Asia	0.932 (0.752–1.156)	0.434	-	-
Europe	1.073 (0.908–1.269)	0.312	-	-
Africa	1.480 (0.633–3.461)	0.269	-	-
Oceania	1.206 (0.612–2.374)	0.509	-	-
Hematological disorders				
TMP group	0.389 (0.315–0.480)	<0.001	0.428 (0.342–0.537)	<0.001
TM group	0.266 (0.209–0.338)	<0.001	0.329 (0.255–0.425)	<0.001
T group	0.088 (0.067–0.116)	<0.001	0.112 (0.083–0.151)	<0.001
Male	0.871 (0.749–1.013)	0.028	0.799 (0.683–0.935)	0.001
Age	0.994 (0.990–0.999)	0.002	-	-
South America	1.570 (1.118–2.203)	0.001	1.673 (1.176–2.380)	<0.001
Asia	2.773 (2.229–3.448)	<0.001	2.108 (1.670–2.660)	<0.001
Europe	3.345 (2.784–4.019)	<0.001	2.547 (2.096–3.096)	<0.001
Africa	2.337 (0.899–6.073)	0.033	2.128 (0.806–5.619)	0.063
Oceania	2.315 (1.141–4.697)	0.005	1.486 (0.718–3.075)	0.193
Drug toxicity				
TMP group	1.104 (0.812–1.501)	0.441	1.289 (0.926–1.795)	0.066
TM group	0.729 (0.524–1.016)	0.023	0.965 (0.675–1.381)	0.814
T group	0.706 (0.514–0.969)	0.009	1.027 (0.725–1.456)	0.852
Male	1.347 (1.140–1.591)	<0.001	1.272 (1.074–1.507)	0.001
Age	0.997 (0.993–1.002)	0.196	-	-
South America	1.093 (0.755–1.582)	0.567	1.235 (0.848–1.799)	0.179
Asia	1.991 (1.589–2.495)	<0.001	1.974 (1.543–2.524)	<0.001
Europe	2.716 (2.267–3.253)	<0.001	2.920 (2.394–3.562)	<0.001
Africa	1.777 (0.639–4.941)	0.178	1.341 (0.389–4.628)	0.570
Oceania	2.612 (1.369–4.980)	<0.001	2.875 (1.493–5.537)	<0.001
Serious adverse events				
TMP group	1.749 (1.142–2.679)	0.002	1.457 (0.882–2.405)	0.072
TM group	3.593 (2.353–5.486)	<0.001	2.759 (1.671–4.557)	<0.001
T group	3.924 (2.587–5.953)	<0.001	2.692 (1.641–4.419)	<0.001
Male	1.363 (1.189–1.564)	<0.001	1.411 (1.199–1.660)	<0.001
Age	1.039 (1.033–1.044)	<0.001	1.036 (1.030–1.042)	<0.001
South America	0.473 (0.358–0.624)	<0.001	0.492 (0.360–0.671)	<0.001
Asia	0.505 (0.417–0.611)	<0.001	0.613 (0.480–0.782)	<0.001
Europe	0.172 (0.138–0.215)	<0.001	0.179 (0.139–0.231)	<0.001
Africa	0.139 (0.025–0.770)	0.006	0.139 (0.012–1.562)	0.051
Oceania	0.073 (0.013–0.402)	<0.001	0.128 (0.023–0.710)	0.004

Appendix I

**Table 10:** Summary of adverse event categories in kidney-only transplant recipients by continent Data are summarized as frequencies (reporting proportions). TMPB, tacrolimus + mycophenolate mofetil + prednisone + basiliximab; TMP, tacrolimus + mycophenolate mofetil + prednisone; TM, tacrolimus + mycophenolate mofetil; T, tacrolimus monotherapy

Continent	Adverse event category	TMPB group	TMP group	TM group	T group
North America		N = 194	N = 2,230	N = 1,922	N = 3,302
	Transplant rejection	39 (20.1)	230 (10.3)	63 (3.3)	108 (3.3)
	Infections	41 (21.1)	375 (16.8)	280 (14.6)	213 (6.5)
	Renal disorders	50 (25.8)	293 (13.1)	168 (8.7)	176 (5.3)
	Pancreatic disorders	3 (1.5)	4 (0.2)	1 (0.1)	7 (0.2)
	Gastrointestinal disorders	33 (17.0)	166 (7.4)	164 (8.5)	190 (5.8)
	Hematological disorders	28 (14.4)	134 (6.0)	75 (3.9)	39 (1.2)
	Drug toxicity	9 (4.6)	147 (6.6)	52 (2.7)	98 (3.0)
	Serious adverse events	10 (5.2)	208 (9.3)	340 (17.7)	552 (16.7)
Asia		N = 354	N = 913	N = 581	N = 736
	Transplant rejection	82 (23.2)	78 (8.5)	38 (6.5)	42 (5.7)
	Infections	71 (20.1)	169 (18.5)	99 (17.0)	39 (5.3)
	Renal disorders	106 (29.9)	165 (18.1)	117 (20.1)	42 (5.7)
	Pancreatic disorders	1 (0.3)	3 (0.3)	1 (0.2)	0 (0.0)
	Gastrointestinal disorders	46 (13.0)	69 (7.6)	46 (7.9)	14 (1.9)
	Hematological disorders	116 (32.8)	76 (8.3)	34 (5.9)	17 (2.3)
	Drug toxicity	21 (5.9)	101 (11.1)	47 (8.1)	29 (3.9)
	Serious adverse events	8 (2.3)	44 (4.8)	66 (11.4)	86 (11.7)
Europe		N = 394	N = 2,298	N = 1,093	N = 995
	Transplant rejection	60 (15.2)	167 (7.3)	90 (8.2)	43 (4.3)
	Infections	91 (23.1)	426 (18.5)	124 (11.3)	65 (6.5)
	Renal disorders	112 (28.4)	475 (20.7)	149 (13.6)	149 (15.0)
	Pancreatic disorders	3 (0.8)	5 (0.2)	0 (0.0)	5 (0.5)
	Gastrointestinal disorders	45 (11.4)	192 (8.4)	82 (7.5)	50 (5.0)
	Hematological disorders	58 (14.7)	298 (13.0)	115 (10.5)	61 (6.1)
	Drug toxicity	35 (8.9)	206 (9.0)	92 (8.4)	153 (15.4)
	Serious adverse events	9 (2.3)	84 (3.7)	22 (2.0)	21 (2.1)
